# Prevalence of *Plasmodium* species in Badr Governorate, Madinah Province, Saudi Arabia using microscopy and rapid diagnostic test

**DOI:** 10.1097/MD.0000000000035516

**Published:** 2023-10-20

**Authors:** Raafat Abdel Moneim Hassanein, Mohammed Othman Alkurbi, Saad Hussain Alsobhi

**Affiliations:** a Department of Laboratory Medicine, Faculty of Applied Medical Sciences, Umm Al-Qura University, Makkah, Saudi Arabia; b Department of Zoonoses, Faculty of Veterinary Medicine, Assiut University, Assiut, Egypt; c Badr General Hospital, Ministry of Health, Badr Governorate, Madinah Province, Saudi Arabia.

**Keywords:** Madinah, malaria, microscopy, rapid diagnostic test, Saudi Arabia

## Abstract

Malaria infection still represents a notable public health risk in Saudi Arabia. This cross-sectional study aimed to determine the prevalence of *Plasmodium* species among clinically suspected cases who presented at Badr General Hospital and healthcare facilities in selected regions of Badr Governorate, Madinah Province, Saudi Arabia between January 2021 and January 2022. A total of 493 suspected patients were recruited from Badr Governorate, investigated for malaria infection using CBFME and rapid diagnostic test- CareStart Malaria Pf/PAN (HRP2/pLDH) Ag Combo rapid diagnostic tests. The results showed that malaria infection was 34 (6.89%) cases among 493 suspected patients using microscopic examination as reference test. Moreover, subjects aged 31 to 40 years and those aged 51 to 60 years had the highest (50%) and lowest (8.82%) percentages of malaria cases. *Plasmodium vivax* (19/34, 55.88%) was higher than *P falciparum* (15/34, 44.1%) as the causative agents of malaria cases. The majority of malaria cases (29/34, 80.9%) among non-Saudi mainly from Sudan (15/34, 44.1%), Pakistan (5/34, 14.7%), Bangladesh (5/34, 14.7%) and India (4/34, 11.76%) whereas malaria cases among Saudis (5/34, 14.7%). In addition, the majority of malaria cases (32/34, 94.11%) among male subjects while (2/34, 5.88%) among females. The current study revealed that malaria transmission is still active in Badr Governorate, Madinah Province, Saudi Arabia and represents a public health concern. Further screening implements and continuous epidemiological monitor of the status of malaria infection in Kingdom of Saudi Arabia are thus warranted to improve its controlling activities and eradicate malaria endemicity in the country.

## 1. Introduction

Malaria still represents a worldwide public health risk that annually threatens the lives of millions of victims especially in developing countries, particularly in tropical and subtropical regions. Over 3.4 billion people worldwide are at risk of malaria infection.^[1]^

*Plasmodium* infection in humans can be caused by one of the following malaria parasites (*P falciparum, P vivax, P malariae, P ovale*, and *P knowlesi*). *P falciparum* causes the most severe form of the disease in tropical areas. In recent years, it has been recognized that *P vivax* is also related to severe symptoms.^[2,3]^

Malaria is a highly significant disease transmitted by female *Anopheles (An*) mosquitoes, primarily through their bites. Additionally, *Plasmodium* parasites can also be transmitted either from a mother to her baby during pregnancy (trans-placentally) or through contaminated blood transfusion from an infected blood donor.^[4,5]^

The Global incidence of malaria is increasing due to 3 main factors that play an important role in disease transmission; factors due to human immunity, migrations to malaria epidemic areas and imported malaria cases into non-endemic countries,^[6–8]^ factors related to the vector increasing breeding possibilities that result from climatic and ecological conditions, such as rainfall patterns, humidity, water reservoirs, presence of plants, change in whether patterns, appearance of new vectors by the continuous broken down of vectors control programs due to lack of quality control and growing insecticide resistance.^[5,9]^ Finally, factors directly related to the parasite, as the appearance of resistance to conventional anti-malarial drugs.^[10]^ Consequently, the levels of transmission risk and incidence of malaria change seasonally at the country and regional levels.^[11,12]^

Thirty-two countries are challenging to eliminate malaria and Kingdom of Saudi Arabia (KSA) is now one of these countries.^[1]^ Generally, malaria transmission in KSA is considered unstable and low compared to areas with high transmission rates like Africa and South-East Asia.^[3,11,13]^ The epidemiological aspects of malaria in KSA vary from 1 year to another and from 1 region to another even within areas of the same region.^[14,15]^ KSA contains many of the world mosquito vectors of parasitic diseases including malaria.^[5]^ The risk of acquisition of malaria in Saudi Arabia is limited to the Southwestern part of the country, with the highest number of cases reported from Jazan and Asir regions.^[5,10,12,14]^ The Western, Saudi Arabia, especially in Makkah and Madinah region are a world destination for religious rituals and work. Most people who visit Makkah and Madinah every year are from malaria endemic countries. The Hajj and Umrah religious rituals may introduce malaria into KSA, as many Muslim countries have a high malaria prevalence.^[6–8,16,17]^

Symptomatic diagnosis alone can be misleading for suspected malaria patients due to many other diseases have similar symptoms to malaria. Malaria symptom is a fever due the toxins release when erythrocytic schizonts rupture. Splenomegaly occurs in all forms of malaria. Anemia and jaundice seen also as features of malaria cases. Since severity of malaria processes depends enormously on the species of *Plasmodium* involved, differential diagnosis is therefore crucial for the disease monitoring.^[1,4,18]^

The World Health Organization strongly advises conducting prompt malaria diagnosis, either through microscopy or rapid diagnostic tests (RDTs), for all patients suspected of having malaria before initiating treatment. Early and precise diagnosis is crucial for effectively managing the disease and ensuring robust malaria surveillance.^[4,19]^

A prompt, accurate diagnosis will facilitate case management and thereby reduce morbidity and mortality.^[1,4]^ Microscopy is the gold standard technique used for decades to diagnose malaria due to its capability to identify *Plasmodium* species and their circulating stages (e.g., trophozoites, schizonts, gametocytes), as well as the quantification of parasitemia levels, with low cost, but it is time consuming, and needs an experienced microscopists and continuous quality control and quality assurance systems.^[12,13,19]^ Malaria antigen rapid diagnostic tests based on antigens detection are useful alternative to microscopy, easy to use, fast and cost-effective. However, Malaria antigen rapid diagnostic tests may not be accurate in low-density or asymptomatic infections as well as those from parasite strains that have deletions in the genes encoding histidine-rich protein (HRP2) or (HRP3), its structural homologue.^[20,21]^

In spite of a progress toward malaria elimination in Saudi Arabia has been investigated by several researchers^[14,17]^ a residue remains in some regions of Saudi Arabia such as Qassim^[7]^ and Western regions of Saudi Arabia.^[5,10,12,14,17,22,23]^ The elimination of malaria in Saudi Arabia requires high-quality surveillance data to quickly detect and respond to individual cases. However, there is a scarcity of information about the frequency and distribution of malaria cases in Badr Governorate, Southwest Madinah Province, Saudi Arabia.Therefore, this study aims to fill this gap in knowledge and to elucidate the situation regarding the level of residual malaria endemicity in this region to assist in malaria prevention efforts in addition to highlight the prevalence of *Plasmodium* species in Badr Governorate using microscopy and RDT among suspected malaria patients between January 2021 and January 2022.

## 2. Materials and methods

### 2.1. Study design and setting

A cross-sectional study was carried out to highlight the prevalence of *Plasmodium* species among clinically suspected cases who presented at Badr General Hospital and healthcare facilities in selected regions from Badr Governorate, Madinah Province, KSA.

**Figure 1.**
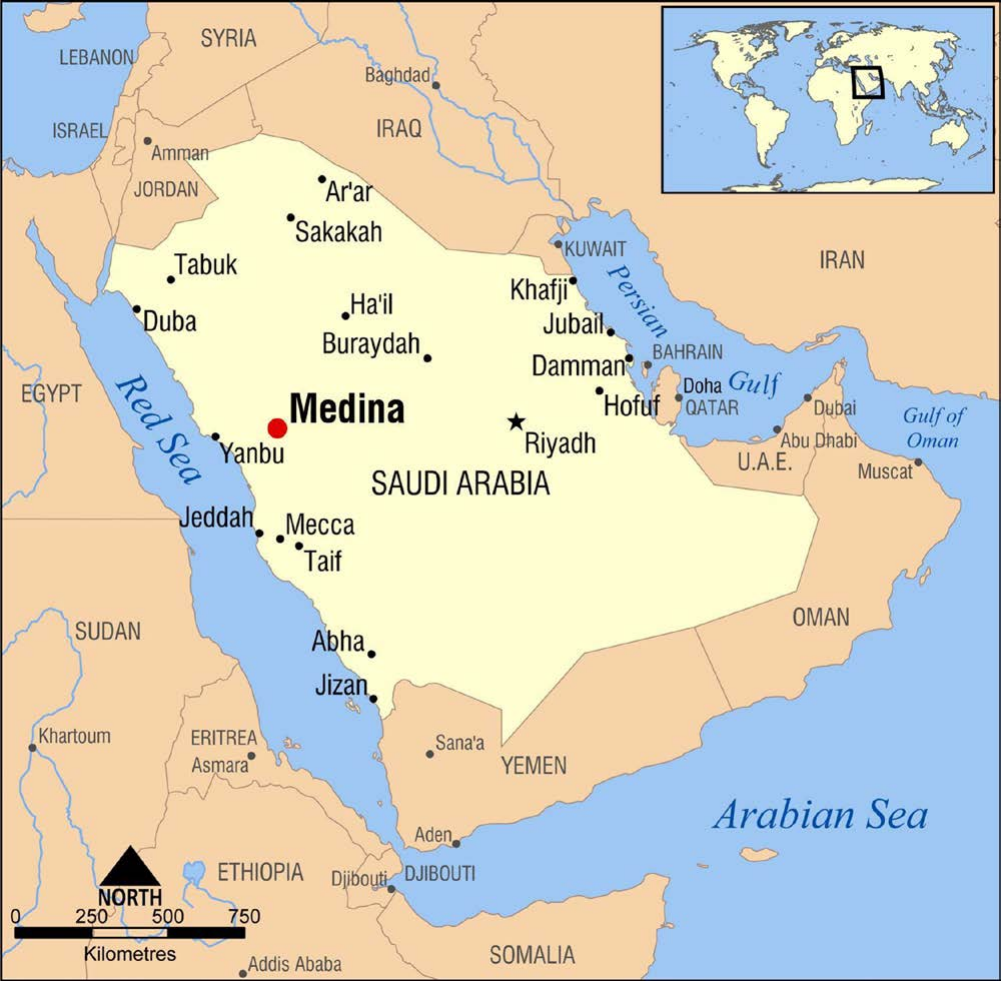
Location map of the Kingdom of Saudi Arabia.

Geographically, Madinah Province, located in the west of the KSA, the area and population (census, 2021) (1,51,99 km^2^, 21,32,679), the average rainfall for Madinah (3 mm) (Fig. 1).

Badr (8226 km^2^, 67,632) is 1 from 8 Governorates belong to Madinah Province, located 130 kilometers southwest of Madinah and lies in a harsh natural landscape of desert plains, steep hills and sand dunes. It includes a city and 91 villages. Badr Governorate is distinguished from other Governorates by the multiplicity of terrain and different environments, including mountainous, desert, agricultural, marine, valleys and plains.

### 2.2. Subjects

Study populations were recruited from clinically suspected cases who presented at Badr General Hospital and healthcare facilities in selected regions of the Badr Governorate. Initially a total of 880 suspected patients were recruited for the study. After the clinical examination of suspected patients with feverish conditions by the physicians of each corresponding hospital and the application of adopted inclusion and exclusion criteria. Inclusion criteria comprised any patient suffering from episodes of fever ≥38^o^C for <10 days followed by episodes of normality, chills, sweating and/or signs of anemia and blood hemolysis. But exclusion criteria included those diagnosed with measles, chickenpox, infected wounds, and pneumonia. A total of 493 patients fulfilled the inclusion criteria were included as suspected malaria patient and served as the study population.

Selected demo-graphic population data were obtained including age, sex and nationality using structured questionnaire. All data were treated as categorical variables in addition to *Plasmodium* species. Age was categorized into age groups of 10-year intervals.

### 2.3. Specimen

Approximately 2 to 3 mL of venous blood was collected from each patient into an ethylenediaminetetraacetic acid tube clearly labeled with the patient reference number, name, sex, and age. Directly after collection, blood samples were examined using a malaria RDT (CareStart Malaria Pf/PAN (HRP2/pLDH) Ag Combo RDT. Thin and thick blood films were prepared on clean, labeled glass slides and stained with diluted Giemsa stain.

### 2.4. Conventional blood film microscopic examination (CBFME)

Conventional blood film microscopic examination (CBFME) by thick and thin blood films were used as standard reference method. The thin and thick blood smears were stained with fresh 10% Giemsa solution screened for malaria parasites by microscopy with (100×) oil immersion magnification as per the World Health Organization protocol.^[19]^ Stained thin film preparations of positive thick films were examined to determine the *Plasmodium* species. Two different microscopists examined each blood film before declaring the slide positive or negative with a senior microscopists reexamined blood films with conflict results. An average of 200 fields were examined before declaring slides negative for malaria parasite.

### 2.5. CareStart Malaria Pf/PAN (HRP2/pLDH) Ag Combo RDT

The CareStart RDT kit (Access Bio, Inc., at Somerset NJ), Cat. No. RMRM-02572, is a rapid test for the qualitative detection of *P falciparum* HRP2 released from *P falciparum* and *Plasmodium* Lactate Dehydrogenase (pLDH), antigen specific to *Plasmodium* species (*P falciparum, P vivax, . malariae*, and *P ovale*). The test with 5µL of whole blood in specimen insertion hole, and then 4 drops of assay buffer were added and wait for 15 to 30 minutes, and then read the results. The presence of a line next to “C” indicate a negative result, while 2 lines (one line in the window next to “C” and another line in the window next to “1”) indicate a positive result for *P falciparum*. The presence of 2 lines (one line in the window next to C and another line in the result window next to “2”) indicate PAN positive (*P falciparum, P vivax, P malariae*, and *P ovale*). The presence of 3 lines (3 lines in the result window next to “C,” “1,” “2”) indicate positive for *P falciparum* or mixed infection for *P falciparum* and other malaria (*P vivax, P ovale*, and *P malariae*). The test is invalid when a line does not appear next to “C.”^[24,25]^

Diagnosis of CareStart RDT kit in comparison to CBFME was evaluated with CBFME results as reference test. Moreover, sensitivity, specificity, positive predictive values, negative predictive values, accuracy index of the CareStart RDT were calculated using CBFME results as reference test.

### 2.6. Ethical Review

Institutional Review Board (IRB # 034-2022) was obtained from the General Director of Health Affairs in Madinah, National Registration Number with NCBE, KSA: (H-03-M-84), Chairman of IBR Committee, ministry of health, Madinah, KSA (Dr Mohammed J. Alkhalawi).

### 2.7. Statistical analysis

Data entry and analysis were done using statistical package for the social sciences software Package version 26.0 (SPSS Inc. Chicago, Illinois) and Microsoft Excel 2010. The Chi-square (χ^2^) test was used for the categorical data analysis. Crosstab was used for determining the frequency distribution of various variables. Finally, the data were presented using appropriate figures and tables. *P* value of < .05 was considered statistically significant.

## 3. Results

### 3.1. Results of microscopic examination for suspected malaria cases according to sociodemographic characteristics

**Figure 2.**
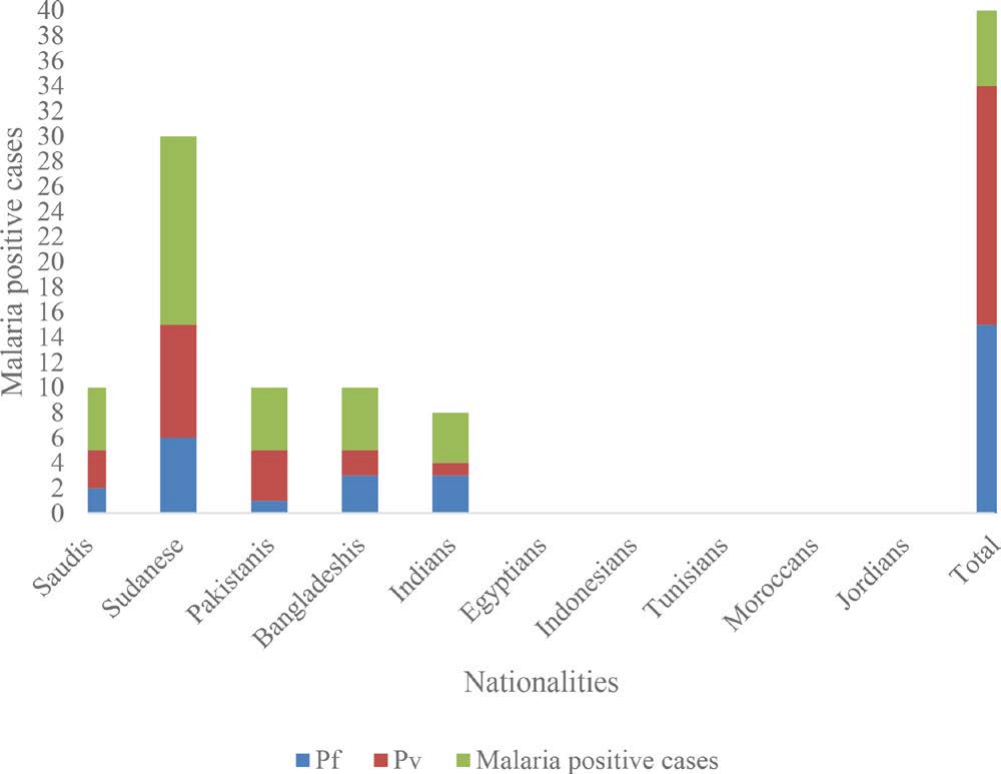
Distribution of *Plasmodium* species in different nationalities in Badr Governorate, Madinah Province, Kingdom of Saudi Arabia (KSA).

Among the 493 tested blood samples, a total of 34 cases (6.89%); were positive-malaria infected patients (Table 1 and Fig. 2). Furthermore, the nationality distribution showed that the highest number of the detected positive cases was in Sudani cases (15/34, 44.11%) while Pakistani (5/34, 14.70%), Bangladeshi (5/34, 14.70%), Saudi (5/34, 14.70%) and Indian (4/34, 11.76%).

**Table 1:** Distribution of malaria cases and their causative *Plasmodium* species in different nationalities in Badr Governorate, Madinah Province, Kingdom of Saudi Arabia (KSA).

Nationalities	Examined patients	Malaria positive cases (%)	Causative *Plasmodium* species	
			Pf	Pv
Saudis	359	5 (14.70%)	2	3
Sudanese	60	15 (44.11%)	6	9
Pakistanis	21	5 (14.70%)	1	4
Bangladeshis	9	5 (14.70%)	3	2
Indians	11	4 (11.76%)	3	1
Egyptians	20	0	0	0
Indonesians	3	0	0	0
Tunisians	8	0	0	0
Moroccans	1	0	0	0
Jordanians	1	0	0	0
Total	493	34	15 (44.1%)	19 (55.88%)

Pf = *Plasmodium* falciparum, Pv = *Plasmodium* vivax.

**Figure 3.**
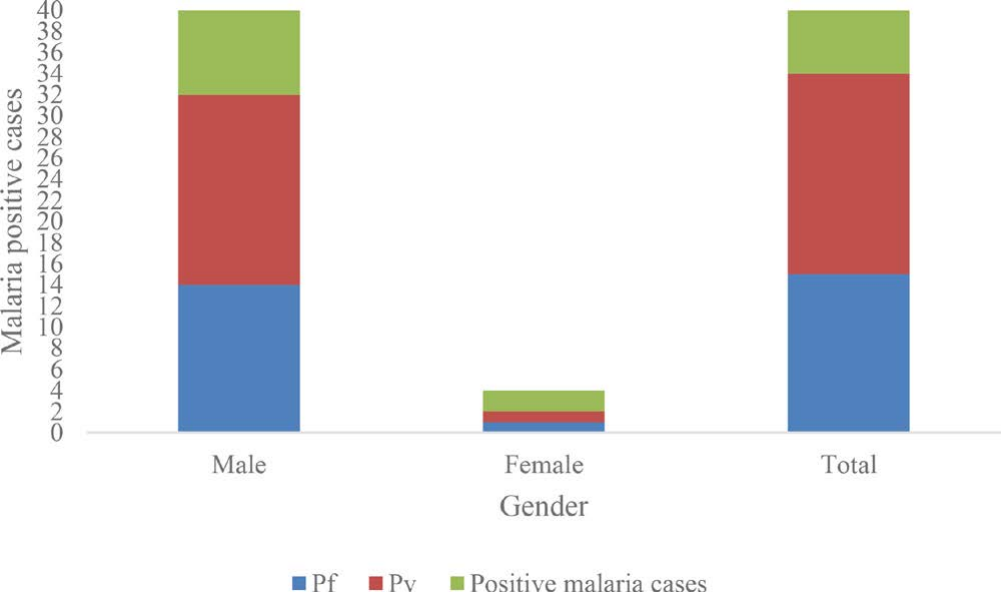
Gender of malarial cases in Badr Governorate, Madinah Province, Kingdom of Saudi Arabia (KSA).

The majority of cases (*P* < .001) (32/34, 94.11%) among male subjects while (2/34, 5.88%) among females (Table 2 and Fig. 3).

**Table 2:** Gender of malarial cases in Badr Governorate, Madinah Province, Kingdom of Saudi Arabia (KSA).

Gender	Results		
	Malaria positive cases (%)	Pf (%)	Pv (%)
Male	32 (94.11)*	14 (41.17)	18 (56.25)
Female	2 (5.88)	1 (2.94)	1 (50.0)
Total	34	15 (44.11)	19 (55.88)

Pf = *Plasmodium falciparum*, Pv = *Plasmodium vivax*.

*P < .001 vs female.

**Figure 4.**
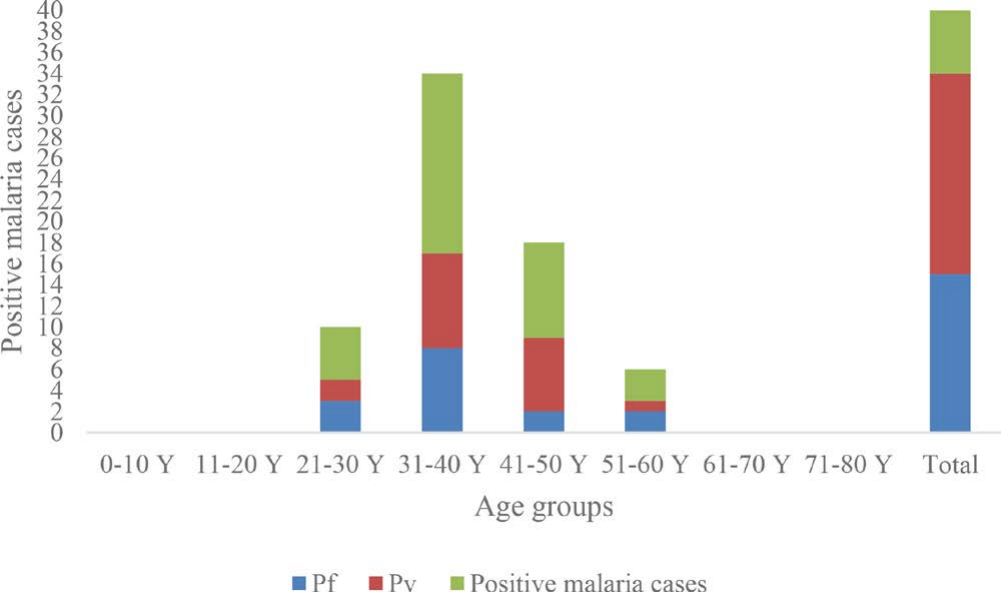
Age group distribution pattern of malarial cases by Badr Governorate, Madinah Province, Kingdom of Saudi Arabia (KSA).

The preponderance of malaria in relation to age was highest (*P* < .05) (17/34; 50%) for ages (31–40 years) as well as those 41 to 50 years demonstrated a pervasiveness of (9/34; 26.47%) whereas the lowest percentage (3/34, 8.82%; *P* < .05) was in age group of 51 to 60 years (Table 3 and Fig. 4).

**Table 3:** Age groups distribution pattern of malarial cases in Badr Governorate, Madinah Province, Kingdom of Saudi Arabia (KSA).

Age group	Examined patients	Results		
		Malaria positive cases (%)	Pf (%)	Pv (%)
0–10 yr	2	0 (0.0)	0	0
11–20 yr	23	0 (0.0)	0	0
21–30 yr	122	5 (14.09)	3	2
31–40 yr	138	17 (50)*	8	9
41–50 yr	111	9 (26.47)	2	7
51–60 yr	55	3 (8.82)	2	1
61–70 yr	34	0 (0.0)	0	0
71–80 yr	8	0 (0.0)	0	0
Total	493	34	15 (44.11)	19 (55.88)

Pf = *Plasmodium falciparum*, Pv = *Plasmodium vivax*, Y = year.

*P < .05 vs other age group sets.

### 3.2. Results of microscopic examination for suspected malaria cases according to *Plasmodium* species

Concerning *Plasmodium* species, *P vivax* was the most frequently detected species (19/34, 55.88%) cases, whereas *P falciparum* (15/34, 44.1%) cases (Tables 1–4 and Figures 2–4).

### 3.3. Results of CareStart RDT kit and microscopy for detection of *Plasmodium* infection among suspected patients

Of the (34/493, 6.89%) microscopically confirmed malaria cases, 19 cases (55.88%) were *P vivax*, 15 cases (44.1%) were *P falciparum*, but 459 suspected patients were negative for *Plasmodium* infection while 29 (5.88%) CareStart RDT kit positives for *Plasmodium* infection in 493 clinically suspected cases, 16 were indicate PAN positive (*P falciparum, P vivax, P malariae*, and *P ovale*), 13 were *P falciparum* but 464 were negative for *Plasmodium* infection. Using CBFME as reference test, CareStart RDT kit showed 5 false-negative samples that were positive by CBFME. Of these 5 negative samples, 3 was *P vivax* and the rest was *P falciparum* on microscopy. Furthermore, CareStart RDT kit showed 16 PAN (*P falciparum, P vivax, P malariae*, and *P ovale*) positive that were *P vivax* positive by microscopy (Table 4.). CareStart RDT kit showed a sensitivity (87.17%), specificity for Pv detection (96.63%), positive predictive value for Pv detection (68%), negative predictive value (98.92%) and accuracy index (98.99%).

**Table 4:** Results of CareStart RDT kit and microscopy for detection of *Plasmodium* infection among suspected patients.

Nationalities	Examined patients	CareStart RDT kit			Microscopy		
		Negative	Pf	PAN positive	Negative	Pf	Pv
Saudis	359	354	2	3	354	2	3
Sudanese	60	46	5	7	45	6	9
Pakistanis	21	16	1	3	16	1	4
Bangladeshis	9	5	2	2	4	3	2
Indians	11	7	3	1	7	3	1
Egyptians	20	20	0	0	20	0	0
Indonesians	3	3	0	0	3	0	0
Tunisians	8	8	0	0	8	0	0
Moroccans	1	1	0	0	1	0	0
Jordanians	1	1	0	0	1	0	0
Total	493	464 (94.11%)	13 (2.63%)	16 (3.24%)	459 (93.10%)	15 (3.04%)	19 (3.85%)

Pf = *Plasmodium* falciparum, PAN positive = (*P falciparum, P vivax, P malariae*, and/or *P ovale*), Pv = *Plasmodium* vivax, RDT = rapid diagnostic tests.

## 4. Discussion

The present study identified 34 (6.89%) malaria cases among clinically suspected cases in Badr Governorate, Madinah Province, Saudi Arabia recruited from January 2021 through January 2022. In support, Amer et al,^[23]^ have reported that Jazan has the highest frequent rate of malaria infection (66.3%) whereas Madinah (16.6%), Makkah (8.7%), Asir (7.4%) and Al-Bahah (1.0%) region, reflecting its endemicity with malaria in the Western regions of Saudi Arabia.

The current study conducted in Badr Governorate in the Western parts of KSA, one of the agricultural governorates, known for its wells, springs as well as presence of Wasit lake (A stagnant lake made of rainwater (, provide an environment for proper breeding sites for the mosquito vectors of malaria (*An* mosquitoes).^[5]^ Regions-related variations in the prevalence of malaria cases was reported worldwide, and such variations have been attributed to various ecological factors including diversity in the rainfall status, groundwater and plants.^[11]^

Based on the assumption made in relation to nationality of study population, the majority of cases 29/34 (80.9%) among non-Saudi. A similar situation was also reported in the neighboring Jazan region.^[14]^ The top nationality with malaria cases in Badr Governorate was Sudanese (15/34; 44.1%), whereas other groups belong to Pakistanis 5/34 (14.7%), Bangladeshis 5/34 (14.7%), Saudis 5/34 (14.7%) and Indians 4/34 (1.0%).

Malaria is endemic in Africa, and in parts of Asia and Americas.^[3,13,24]^. Moreover, it is widespread in developing countries due to the absence of available treatments, effective methods to vector and parasite control as well as the spread of drug and pesticide resistance.^[9,10]^

In Saudi Arabia, imported malaria remains a major problem with a constant flow of imported malaria, mostly among immigrant workers from south Asia (Pakistan, India and Bangladesh), East Africa (Sudan and Ethiopia), and Yemen.^[6–8]^

In the current study, a total of 493 suspected patients were tested; CBFME identified 34 (6.89%) while CareStart RDT kit identified 29 (5.88%). Moreover, Using CBFME as reference, CareStart RDT kit failed to detect 5 positive cases that were confirmed by microscopy as *P vivax* (3) and *P falciparum* (2). False negative results reflecting a missed diagnosis that may lead to patients going untreated and becoming parasite carriers and malaria reservoirs in their communities.^[26]^ False negative results of CareStart RDT kit were attributed to insufficient detecting low-density infections as well as those from parasite strains that have deletions in the genes encoding HRP2, its structural homologue.^[21]^

Moreover, CareStart RDT was indicate 16 PAN positive (*P falciparum, P vivax, P malariae*, and *P ovale*) while it was confirmed as *P vivax* by microscopic examination. The possible explanations for discrepancies in test results obtained by CareStart RDT and microscopic examination due to microscopic examination of Giemsa-stained thick or thin blood smears remains the superior diagnostic method for identifying species and stages of the *Plasmodium* parasites.^[12,19]^ The accuracy of malaria diagnostics depends on several factors, including the level of malaria endemicity, parasite density, mutation or deletion of the gene encoding the HRP2, format and type of the RDT product, and storage conditions.^[13,21]^

RDTs have been developed especially for their ease of use in remote settings in endemic countries.^[12,13]^ However, many drawbacks have been reported with RDTs, especially relating to their sensitivity. In the current study, CareStart RDT kit showed a sensitivity (87.17%) when compared to gold standard microscopy. In agreement, in a field study performed in unstable malaria transmission, performance of CareStart RDT has shown a sensitivity of 85.6% when compared to gold standard microscopy; the sensitivity increased with increasing parasite densities.^[27]^ On contrary, considering PCR as the gold standard, CareStart RDT showed high sensitivity (97.3%) comparable to that performed by expert microscopist 93.2% in a malaria low transmission area of Senegal.^[24]^

Concerning *Plasmodium* species, *P vivax* has also a wide worldwide distribution.^[3]^ The majority of malaria cases that were diagnosed in the current study due to infection with *P vivax* infection, followed by those caused by *P falciparum.* These findings have also been reported previously in Saudi Arabia^[23]^ as well as India,^[28]^ China.^[29]^ In contrary, the previous reports documented *P falciparum* as the main causative agent of malaria in Saudi Arabia.^[8,14]^ Furthermore, the predominant of *P falciparum* compared to other *Plasmodium* species causing malaria was reported in the African, Southeast Asia and Eastern Mediterranean regions.^[13,28,30]^

Moreover, in the current study, subjects aged 31 to 40 years and those aged 51 to 60 years had the highest (50%) and lowest (8.82%) percentages of malaria cases. These findings are constant with the previous reports described the distribution and prevalence patterns of malaria in KSA^[14,23]^ and those of neighboring Gulf countries.^[31,32]^ In addition, a report of World Health Organization^[1,4]^ stated that all age groups are at malaria infection risk but the most deaths in Africa occur in young children. This observation in the current study may be attributed to the fact that individuals with age of 31 to 40 years and above are the most productive and involved in different activities, and this in turn may increase their chances for bitten by malaria infected *An* mosquitoes.

Female subjects showed a lower malaria prevalence (2/34, 5.88%) compared to males (32/34, 94.11%) in the currents study. This observation was also reported in Saudi Arabi^[14]^ as well as in many countries such as Nigeria,^[13]^ Malaysia,^[3,33]^ and India.^[11]^ The observed gender-based distribution among malaria patients could be attributed to the fact that the males are more exposed to the parasite insect vectors as a result of their occupational activities.^[11]^

The current study show that malaria is a disease in the Badr Governorate, Southwest Madinah Province, Saudi Arabia, consequently it needs the development of local prevention plans to minimize the disease occurrence. Due to adult males are at risk because of agriculture related activities or activities outside the house at optimum mosquito-biting hours, this may be a group that needs to be targeted with preventive measures, e.g. insecticide creams for use as topical repellents in the evening or early morning as well as outdoor insecticide spraying.^[11]^

Limitations of the current research include first, there is a lack of data about the clinical presentation and occupation of the positive cases and residency (Urban or rural). Second, the malaria surveys only in Badr Governorate. Finally, no PCR tests were conducted for malaria diagnosis in spite of PCR is highly sensitive and specific for malaria diagnosis.

We suggest that researchers need to conduct a similar study across multiple regions in Saudi Arabia. This approach would help reduce misdiagnosis, thereby providing a more accurate understanding of the epidemiological trends of malaria.

## 5. Conclusions

Data of the present study reveal that despite the efforts exerted for controlling malaria infection in KSA, it remains endemic and represents a public health concern in some regions of KSA particularly Badr Governorate, Madinah Province. Furthermore, combination of RDT and microscopy together with the evaluation of malaria RDTs over time should be a powerful tool for diagnosing malaria in endemic countries. However, further studies to evaluate malaria diagnostics among asymptomatic individuals are required using molecular techniques such as PCR. Moreover, infection with *P vivax* was the predominant type of the detected malaria cases, and the 31 to 40 years age group was the most vulnerable age during the period of the study. Men are at higher risk. In malaria elimination settings, such information is crucial to identify the challenges and further research need towards the elimination of malaria in the targeted areas. Malaria control and elimination will need insecticide spraying to eliminate remaining vector foci. Further screening implements and continuous epidemiological monitor of the status of malaria infection in KSA are thus warranted to improve its controlling activities and eradicate its endemicity in the country.

## Acknowledgments

The authors would like to thank Institute of Scientific Research and Revival of Islamic Heritage at Umm Al-Qura University (Project # 43409024) for the financial support.

## Author contributions

**Conceptualization:** Mohammed Othman Alkurbi.

**Data curation:** Saad Hussain Alsobhi.

**Investigation:** Saad Hussain Alsobhi.

**Methodology:** Saad Hussain Alsobhi.

**Project administration:** Raafat Abdel Moneim Hassanein.

**Resources:** Saad Hussain Alsobhi.

**Software:** Raafat Abdel Moneim Hassanein.

**Supervision:** Raafat Abdel Moneim Hassanein, Mohammed Othman Alkurbi.

**Validation:** Mohammed Othman Alkurbi.

**Visualization:** Mohammed Othman Alkurbi.

**Writing – original draft:** Raafat Abdel Moneim Hassanein.

**Writing – review & editing:** Raafat Abdel Moneim Hassanein.
